# The Developmental Toxicity and Endocrine-Disrupting Effects of Fenpropathrin on *Gobiocypris rarus* during the Early Life Stage

**DOI:** 10.3390/toxics11121003

**Published:** 2023-12-08

**Authors:** Lei Wang, Jinlin Jiang, Jianwei Lu, Tao Long, Yang Guo, Shunan Dong, Huiyi Wu

**Affiliations:** 1 Key Laboratory of Soil Environmental Management and Pollution Control, Nanjing Institute of Environmental Sciences, Ministry of Ecology and Environment, Nanjing 210042, China; 2 College of Agricultural Science and Engineering, Hohai University, Nanjing 210098, China

**Keywords:** fenpropathrin, *Gobiocypris rarus*, early life stage, developmental toxicity, endocrine disruption

## Abstract

In the present study, the developmental toxicity and endocrine-disrupting effects of fenpropathrin on *Gobiocypris rarus* during the early life stage were studied using a semi-static water exposure method. The results showed that the LOEC (lowest observed effect concentration) of fenpropathrin on the incubation of rare minnow embryos was above 2.5 μg·L^−1^. The LOEC and NOEC (no observed effect concentration) of fenpropathrin on the developmental malformations and death indicators were 2.0 and 1.5 μg·L^−1^, respectively. After exposure to 1.5 μg·L^−1^ of fenpropathrin for 31 days, the expressions of androgen receptor genes (*AR*) and sex hormone-synthesis-related genes (*CYP17* and *CYP19a*) were significantly decreased and the expressions of thyroid hormone receptor genes (*TRβ*) and aryl hydrocarbon receptor genes (*AhR1a* and *AhR2*) were significantly increased in juvenile *Gobiocypris rarus*. The expression levels of the androgen receptor gene (*AR*), estrogen receptor gene (*ER1*), and the sex hormone-synthesis-related genes (*HMGR*, *CYP17*, and *CYP19a*) were significantly decreased, while the estrogen receptor gene (*ER2a*), thyroid hormone receptor gene (*TRβ*), and aromatic hydrocarbon receptor genes (*AhR1a* and *AhR2*) were upregulated in juvenile *Gobiocypris rarus* under exposure to 2.0 μg·L^−1^ of fenpropathrin. Relatively low concentrations of fenpropathrin can affect the expression of sex hormone receptor genes, genes related to sex hormone synthesis, thyroid hormone receptor genes, and aromatic hydrocarbon receptor genes, thus interfering with the reproductive system, thyroid system, and metabolic level in *Gobiocypris rarus*. Therefore, more attention should be paid to the endocrine-disrupting effect caused by the pyrethroid insecticides in the water environment. Furthermore, studies on the internal mechanism of the endocrine-disrupting effect of pyrethroid insecticides on fish is needed in the future.

## 1. Introduction

Pyrethroid pesticides are a type of highly efficient insecticide that have been used worldwide for a long time. Due to their strong hydrophobicity [1], they can easily accumulate in aquatic organisms and are highly toxic to aquatic organisms, so they pose certain risks to aquatic ecosystems. Fenpropathrin is one of the types of pesticides used to replace organochlorine pesticides, and its insecticidal toxicity is more than 10 times higher [2]. Fenpropathrin is a pyrethroid insecticide with a contact-killing effect, stomach toxicity, and certain repellent effects. It can also act as a nerve agent because of its high ester solubility. Fenpropathrin is mainly used to prevent and control a variety of diseases in cotton, apple, cabbage, and other crops. Although it has the characteristics of high efficiency, low toxicity, and low residue, it still poses harm to the environment and ecology. Fenpropathrin, also known as Meothrin, is a pyrethroid pesticide that acts on the nervous system and can damage it. Pyrethroid pesticides are widely used in insect spectra because of their common properties of contact and gastric toxicity. Such pesticides act quickly and aquatic organisms are very sensitive to them. This kind of pesticide has an irritating effect, gastrointestinal effects, and acute effects, and once consumed by people, it may cause disease, deformity, cancer, and mutation. Even if symptoms do not occur immediately, toxicity is stored in the body and may double. Therefore, fish poisoned by such pesticides cannot be eaten by humans [3]. In general, pyrethroid pesticides are metabolized quickly in mammals and higher vertebrates and can be quickly eliminated, but they have a lower metabolism and excretion capacity in aquatic organisms and a higher toxicity [3,4]. As the first country to use pyrethroid pesticides, the United States has been paying attention to the impact of pyrethroid pesticides on the aquatic ecological environment for a long time [4]. Due to the wide application of pyrethroid pesticides, their presence has been detected in an increasing number of water environments around the world in recent years [4,5,6,7,8,9,10,11]. Due to the large number of such pesticides in China, the remaining pesticides in the water environment may cause harmful effects on aquatic organisms. Acute studies have shown that most pyrethroids are highly toxic to plankton, such as cladocerans, shrimp, and fish, and on the whole, the toxicity towards the first two (cladocerans and shrimp) is higher than that of the latter (fish). For example, the LC_50_ of fenpropathrin against honeybee larvae was 15.120 mg·L^−1^ [10,11]. The 24 h LC_50_ of fenpropathrin against loach was 85.51 μg·L^−1^ [12]. The 96 h LC_50_ of fenpropathrin against *Megalobrama hoffmanni* was 1.866 μg·L^−1^ [13]. The 24 h LC_50_ of fenpropathrin to Qiandongnan field fish (carp) was 28.480 μg·L^−1^ [14]. The 96 h LC_50_ of fenpropathrin against blood clam was 286.5 ng·mL^−1^ [15]. Fenpropathrin represents a class II pyrethroid insecticide and its main toxicological effects in target organisms are neuronal and cardiovascular. However, a screening by Padilla et al. in 2012 revealed developmental effects with an AC50 of 0.32 µM in zebrafish (Danio rerio) [16]. The above research indicated that the risk of pyrethroid pesticides to water ecology should not be ignored. At present, there are relatively few studies on the toxicity mechanism and toxic effect characteristics of pyrethroids on aquatic organisms, and studies have shown that exposure to fenpropathrin may have certain effects on the immune system, detoxification system, and intestinal flora composition of Italian bees [17]. Other studies have shown that fenpropathrin can significantly promote the absorption of galiquiquone in rats, which may be related to damage in the small intestine tissue and a reduction in tight junction protein expression [18,19]. *Car Es*, *MFOs*, and *GSTs* have detoxification effects in LC_10_ and LC_20_ fenpropathrin-treated codling moths, but *Car Es* plays a dominant role [20]. In addition, many studies have revealed that fenpropathrin has a significant enrichment effect in zebrafish, which is concentration dependent, can also lead to a variety of teratogenic effects in the development of embryo yolk sac, and can also produce an obvious thyroid interference effect in zebrafish [21].

*Gobiocypris rarus* is a freshwater fish species endemic to China, mainly living in tributary ditches and other small water bodies, and is distributed in Hanyuan County, Sichuan Province, China. *Gobiocypris rarus* has the advantages of short sexual cycles, easy feeding, and sensitivity to chemical substances and is an ideal material for chemical toxicity tests and environmental water sample toxicity experiments [14,22]. In the present study, *Gobiocypris rarus* is selected as the trial organism to study the developmental toxicity and endocrine-related nuclear receptor gene expression interference effects of fenpropathrin exposure at the early life stage, laying a foundation for studies on the characteristics and mechanisms of the toxicity effects of abundant fenpropathrin in aquatic organisms as well as a more complete aquatic ecological risk assessment.

## 2. Materials and Methods

### 2.1. Chemicals

Fenpropathrin (92.9%), purchased from Jiangsu Nongbo Biotechnology Co., LTD, Yancheng, China; DMSO, purchased from Shanghai Lingfeng Chemical Reagent Co., LTD, Shanghai, China; liquid nitrogen; RNase-Free ddH_2_O; RNAprep Pure Tissue Kit (TIANGEN, Beijing, China); HiScript III RT SuperMix for qPCR (Vazyme, Nanjing, China, R323-01); and ChamQ Universa l SYBR qPCR Master Mix (Vazyme, Q711-02).

### 2.2. Subject Materials

The test fish species is a rare minnow (*Gobiocypris rarus*). The species is an IHB closed group. Breeding fish were obtained from the Institute of Aquatic Biology, Chinese Academy of Sciences, and the introduction lot number is rare minnow 20180728. Healthy, undamaged, and robust adult fish were selected from the batch as parents, and they were housed in a recirculating culture system in the laboratory and domesticated for 14 d under the following conditions: temperature (25 ± 2) °C, 14 h of light per day, dissolved oxygen greater than 60% of the saturated concentration in air, and regular feeding of fairy shrimp twice daily (08:00 and 16:00).

The male and female rare minnow parents were paired in a 1:1 ratio, and the behavior of male and female fish was observed daily. When the male fish were observed to chase the female fish, the male and female fish were retrieved and the fertilized eggs were obtained via artificial fertilization. The eggs were randomly sampled under a microscope to confirm that the fertilized eggs were in the unicellular stage before the test started.

The test water used was tap water treated with aeration for more than 24 h. The water hardness was 145.0 mg·L^−1^ (in terms of CaCO_3_) and the pH was 7.82. The relevant water quality parameters were entrusted to Jiangsu Provincial Center for Disease Control and Prevention for determination.

### 2.3. Exposure Experiment

#### 2.3.1. Test Solution Preparation

An amount of 0.05382 g of the fenpropathrin technical material (92.9%) was accurately weighed and dissolved with 20 mL of DMSO. The solution was increased to a total volume of 500 mL with distilled water to obtain a concentration of 100 mg·L^−1^ stock solution. When used, the stock solution was further diluted to a solution of 1 mg·L^−1^, and then the treatment group solution was prepared by diluting the solution to a concentration of 0.5, 1.0, 1.5, 2.0, and 2.5 µg·L^−1^. The concentration of 0.1 mL·L^−1^ DMSO solution was set as the solvent control group. The test solution was renewed every 72 h.

#### 2.3.2. Fish Early Life Stage Toxicity Test

The concentrations of fish in the early life stage toxicity gradient test were set at 0.5, 1.0, 1.5, 2.0, and 2.5 µg·L^−1^, while a blank control group without the test substance and a control group with dimethyl sulfoxide (DMSO) solvent were also set. The stability results for the test solution showed that, under the semi-static test conditions (water change frequency of 72 h), the change in the test concentration during the test was less than ±20% of the initial test concentration, so the test was conducted using the semi-static test method.

During the test, the volume of the tested solution was 1 L, and the loading rate of the solution in the container did not exceed 5 g·L^−1^. Each concentration group and control group had 90 eggs. There were three parallel groups. The test was started after the eggs were fertilized and the embryos were immersed in the test solution before the embryonic disc began to divide. The incubation period was 3 days, 28 days after hatching, for a total of 31 days. The light/dark ratio was 14 h:10 h, the temperature was 24.2~25.8 °C, and the dissolved oxygen concentration was 81.8~96.8%. Parental fish were fed with shrimp, newborn fish were fed with paramecium, and juvenile fish were fed with halogen larvae that had been hatched for 48 h. The first feeding started on the 6th day after spawning. The test container was cleaned each time the test solution was changed. The stage of embryo development was accurately identified at the beginning of subject exposure. Egg hatching and survival of the young were observed once a day during the experiment and the number was recorded. Dead individuals (eggs, embryos, or smolts) were removed promptly. It was important to be very careful when removing them so as not to hit or damage the surrounding eggs or fish larvae. The number of deformed or discolored hatchlings was recorded daily, and the natural incidence of abnormal embryos and hatchlings in the control group was recorded. The deformed individuals were removed when they died. Abnormal movements, such as rapid breathing, swimming disorders, abnormal resting, and abnormal feeding, were recorded daily.

### 2.4. Fluorescence Quantitative PCR Experimental Method

After 31 d of exposure, the rare minnow juveniles were snap-frozen in liquid nitrogen at each treatment concentration and the total RNA was extracted from the control group according to the instructions for the RNA extraction kit. Then, the concentration and purification quality of RNA were determined using a nucleic acid protein meter (Beckman-DU800, Focos Technology Co., LTD, Nanjing, China), and the RNA was reverse-transcribed according to the instructions for the reverse transcription kit to obtain complementary DNA (cDNA). Finally, 20 µL of the reaction system was prepared on ice according to the instructions for the real-time fluorescence PCR kit and the following reaction program was performed: 95 °C pre-denaturation for 30 s; 95 °C for 10 s, 60 °C for 30 s, 40 cycles; the melting curve acquisition program used the default acquisition program of the fluorescence quantitative PCR instrument (Bio-Rad CFX96^TM^Real-Time System, Bole Life Medical Products (Shanghai) Co., LTD, Shanghai, China). The primer sequences of the genes used are shown in Table 1.

### 2.5. Data Processing

The relative gene expression was calculated using the 2^−ΔΔCt^ method, where ΔΔΔCt was calculated using Equation (1):

ΔΔCt = (Ct_target_ − Ct_actin_)·t_x_ − (Ct_target_ − Ct_actin_)·t_0_ (1)

where Ct denotes the cycling threshold; Ct_target_ denotes the Ct value of the target gene; Ct_actin_ denotes the Ct value of the internal reference gene; t_x_ denotes an arbitrary time point; and t0 denotes the target gene expression at 1-fold amount corrected using the β-actin gene.

There were five concentration treatment groups (0.5, 1.0, 1.5, 2.0, 2.5 µg/L), as well as one blank control group and one solvent control group. Each of these seven groups has three parallel groups. Under the preconditions of normal distribution and Chi-square, one-way ANOVA and least significant difference (LSD) test were used to analyze the significance of differences between the treatment and control groups (*p* < 0.05 was considered significant, denoted by *); otherwise, non-parametric tests were used to test the significance of differences between groups, and the results were expressed as mean ± standard error (SE). Plots were created with Origin 9.1 software.

## 3. Results and Analyses

### 3.1. Effects of Fenpropapathrin on Embryo Hatchability of Gobiocypris rarus

**Figure 1:**
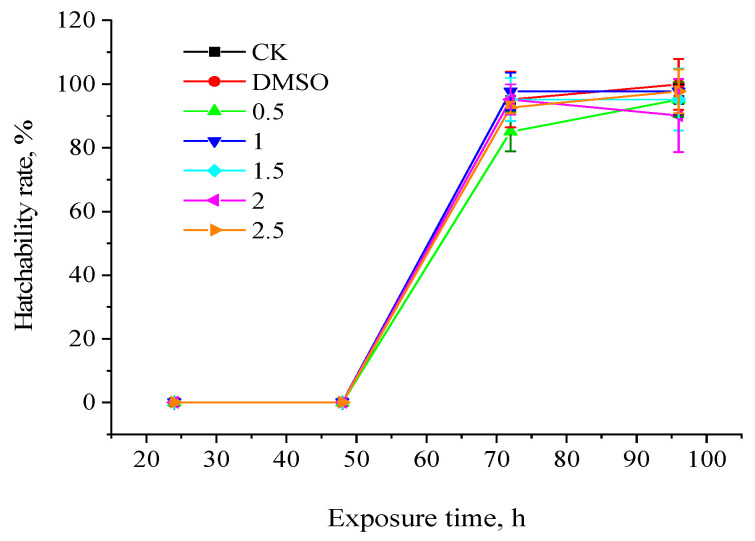
Effects of fenpropathrin on embryo hatchability of *Gobiocypris rarus.* All data are shown as mean ± SE (n = 3).

Figure 1 displays the outcomes of the experiment. It is evident that *Gobiocypris rarus* embryos progressively started to hatch 72 h after incubation and finished the process at 96 h. Fenpropathrin at concentrations of 0.04–3.00 μg·L^−1^ does not significantly affect the incubation of *Gobiocypris rarus*, as indicated by the significant difference analysis, which also found no significant difference between the incubation rates of each concentration group and the control group.

### 3.2. Effects of Fenpropapathrin on the Rate of Deformability in the Early Life Stages of Gobiocypris rarus

**Figure 2:**
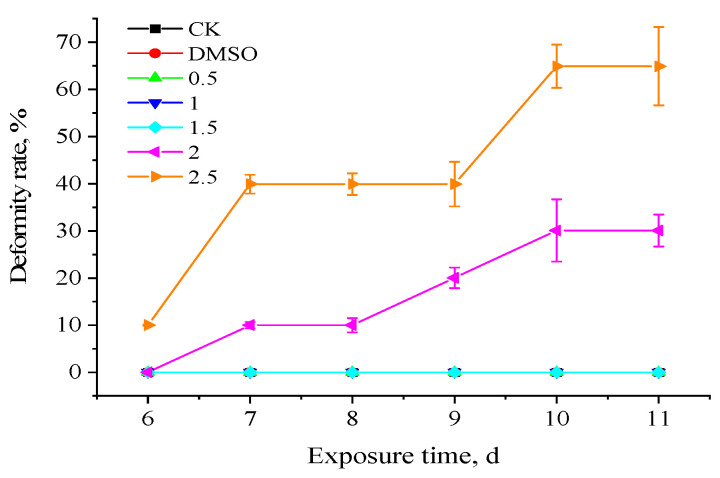
Effects of fenpropathrin on the deformability rate of *Gobiocypris rarus.* All data are shown as mean ± SE (n = 3).

**Figure 3:**
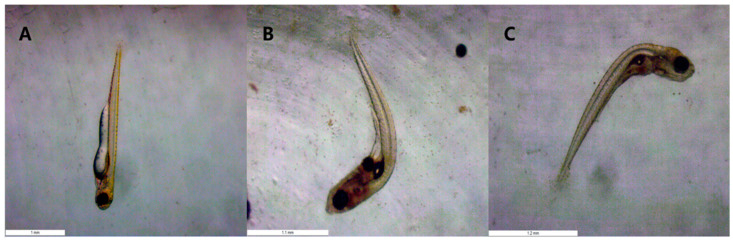
Teratogenic effect of fenpropathrin on the larvae of Chinese rare minnow. All data are shown as mean ± SE (n = 3). (**A**): Normal larva (CK, 168 h); (**B**): shaft bending (2.0 µg/L, 168 h); (**C**): shaft bending (2.5 µg/L, 168 h).

It was discovered during the experiment that fish larvae in the 2.5 µg·L^−1^ concentration group started to exhibit abnormalities after 6 days. Fish larvae in the 2.0 µg·L^−1^ concentration group started to exhibit abnormalities after 7 days. No larval deformity was observed in the other concentration groups. The deformed larvae started to die on day ten. Consequently, each concentration group’s variations in malformations from 6 to 11 days were statistically examined, as Figure 2 illustrates. In contrast to broilers exposed to lower quantities in the other groups, which showed no discernible change from the control group, deformities considerably increased in the 2.0 µg·L^−1^ and 2.5 µg·L^−1^ concentration groups. As seen in Figure 3, the primary teratogenic effect of fenpropathrin on Chinese rare minnow was exhibited as axial bending.

### 3.3. Effects of Fenpropapathrin on Early Life Stage Mortality in Gobiocypris rarus

**Figure 4:**
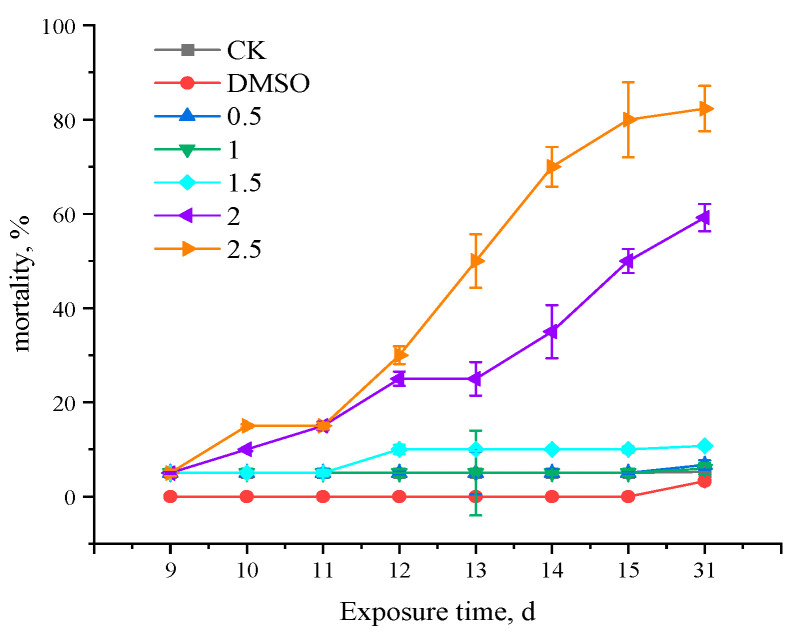
Mortality of *Gobiocypris rarus* broiler fish exposed to fenpropathrin for 9 to 31 days. All data are shown as mean ± SE (n = 3).

Fish with deformities started to die on day 10, and after 15 days there were no more deaths. Figure 4 displays the findings of the calculation of the *Gobiocypris rarus* death rate, which was combined with the malformation statistics. Figure 4 displays the statistics on the *Gobiocypris rarus* 31 d mortality rate that were obtained after the study was completed. The findings indicated that *Gobiocypris rarus* mortality increased significantly in the 2.0 µg·L^−1^ and 2.5 µg·L^−1^ concentration groups, but larval mortality in the other groups exposed to lower concentrations did not differ significantly from the control group.

### 3.4. Effects of Fenpropapathrin on the Expression of Sex Hormone Receptor Genes and Synthesis-Related Genes in Gobiocypris rarus

**Figure 5:**
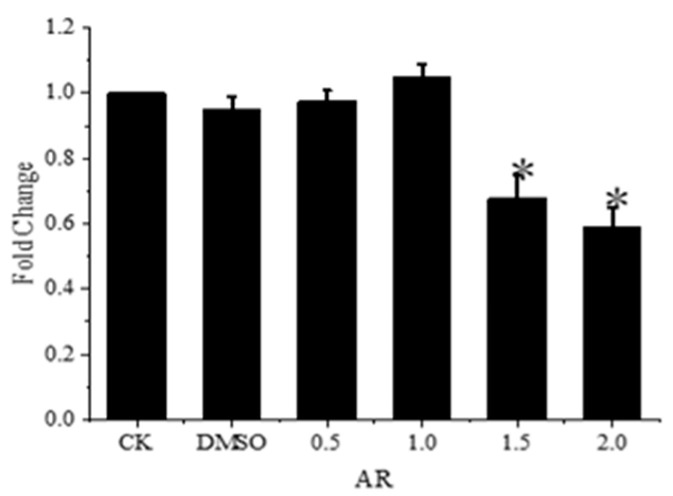
Relative expression of *AR* in young *Gobiocypris rarus* fish after 31 days of fenpropathrin exposure. All data are shown as mean ± SE (n = 3), “*” indicate significant differences (*p* < 0.05).

The research objects chosen to investigate the mechanism of reproductive endocrine disruption of fenpropathrin on the development of *Gobiocypris rarus* are androgen receptors (*AR*) and estrogen receptors (*ERs*). Following a 31-day exposure to fenpropathrin, Figure 5 illustrates a significant decrease in the relative expression level of the *AR* gene in *Gobiocypris rarus* in the 1.5 and 2.0 μg·L^−1^ concentration groups when compared to the control group. These concentration levels were 0.68 and 0.59 times lower, respectively, than those in the control group. When comparing the young *Gobiocypris rarus* in the 0.5 and 1.0 μg·L^−1^ concentration groups to the control group, there was no discernible variation in the relative expression of the *AR* gene.

**Figure 6:**
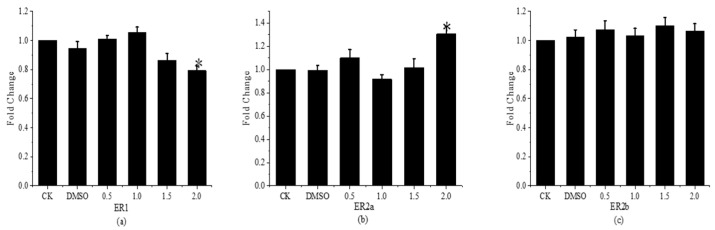
Relative expression of *ERs* in *Gobiocypris rarus* larvae 31 days after fenpropathrin exposure. All data are shown as mean ± SE (n = 3), ”*" indicate significant differences (*p* < 0.05). (**a**): Relative expression of *ER1*; (**b**): Relative expression of *ER2a*; (**c**): Relative expression of *ER2b*.

The findings of the analysis of fenpropathrin’s *ERs* expression in developing fish are displayed in Figure 6. The findings demonstrated that the *ER1* gene’s relative expression level in *Gobiocypris rarus* in the 2.0 μg·L^−1^ concentration group was significantly lower than that of the control group—0.79 times lower—while the *ER2a* gene’s relative expression level was significantly higher—1.31 times higher than that of the control group—in young *Gobiocypris rarus* in the 0.5, 1.0, and 1.5 μg·L^−1^ concentration groups. There was no discernible variation in the relative expression of the *ER2a* gene when compared to the control group. The *ER2b* gene’s relative expression level was not significantly affected by any of the concentration groups.

This study finds that fenpropathrin inhibits the expression of *Gobiocypris rarus*’ androgen receptor genes by comparing and analyzing its effects on the developing sex hormone receptor genes in the species. Fenpropathrin increases the expression of *ER2a* and suppresses the expression of *Gobiocypris rarus ER1* in relation to estrogen receptor genes.

**Figure 7:**
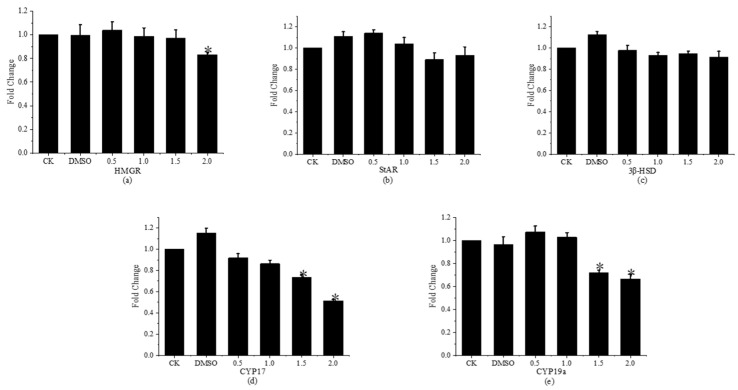
Relative expression levels of genes related to sex hormone synthesis in young *Gobiocypris rarus* fish after 31 days of fenpropathrin exposure. All data are shown as mean ± SE (n = 3), “*” indicate significant differences (*p* < 0.05). (**a**): Relative expression of *HMGR*; (**b**): Relative expression of *StAR*; (**c**): Relative expression of *3β-HSD*; (**d**): Relative expression of *CYP17*; (**e**): Relative expression of *CYP19a*.

Sex hormone metabolism involves a number of enzymes, such as *HMGR*, *StAR*, *3β-HSD*, *CYP17*, and *CYP19a*. Figure 7 displays the relative expression levels of genes associated with sex hormone synthesis in *Gobiocypris rarus* following a 31-day exposure to fenpropathrin.

In immature *Gobiocypris rarus*, the relative expression of the *HMGR* gene was 0.82 times higher in the 2.0 μg·L^−1^ concentration group than in the control group, showing a substantial drop compared to the latter. When comparing the concentration groups to the control group, there was no discernible variation in the relative expression level of the *HMGR* gene in *Gobiocypris rarus*. The expressions of the *StAR* and *3β-HSD* genes were not significantly affected by the fenpropathrin doses of 0.5, 1.0, 1.5, and 2.0 μg·L^−1^. In comparison to the control group, the relative expressions of the *CYP17* and *CYP19a* genes in *Gobiocypris rarus* in the 1.5 and 2.0 μg·L^−1^ concentration groups were considerably lower by 0.74 and 0.51, and 0.72 and 0.66 times, respectively. Young *Gobiocypris rarus* in the other concentration groups had similar relative expressions of the *CYP17* and *CYP19a* genes compared to the control group. The two groups did not differ significantly from one another.

In addition to being a crucial rate-limiting enzyme in the synthesis of cholesterol, *HMGR* is involved in other vertebrate endocrine pathways. Star is a cholesterol transporter that is extensively present in the gonads, adrenal glands, and other animal tissues. It is primarily responsible for the transfer of cholesterol from the outer membrane to the inner membrane of mitochondria. *3β-HSD* is primarily engaged in the catalytic synthesis and conversion of pregnenolone to progesterone, a protein that binds to membranes and is primarily found in the inner membrane of mitochondria. As the primary enzyme in the production of androgens, *CYP17* is in charge of the synthesis of dehydroepiandrosterone (*DHEA*). As an aromatase, *CYP19a* regulates the conversion of testosterone to estradiol and is crucial to the synthesis of steroid hormones. Fenpropathrin decreased the expression of bio-rare *HMGR* in this study, which consequently decreased the synthesis of cholesterol and had a major impact on the synthesis of downstream hormones. A reduced expression of *CYP17* results in suppressed synthesis of 17α-hydroxy-pregnenolone and 17α-hydroxy-progesterone, decreased *DHEA* activity, and ultimately decreased androstenedione yield. Lowering *CYP19a* expression will have an impact on the production of steroid hormones and prevent testosterone from converting to estradiol.

### 3.5. Effects of Fenpropapathrin on Thyroid Hormone Receptor Gene Expression in Gobiocypris rarus

**Figure 8:**
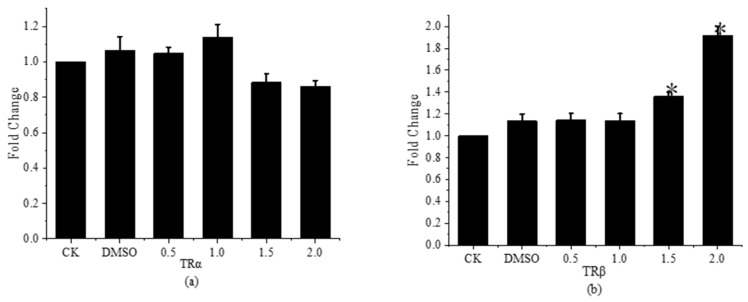
Relative expression levels of *TRs* in young *Gobiocypris rarus* after 31 days of fenpropathrin exposure. All data are shown as mean ± SE (n = 3), “*” indicate significant differences (*p* < 0.05). (**a**): Relative expression of *TRα*; (**b**): Relative expression of *TRβ*.

Within the nuclear receptor superfamily, which is mostly made up of the *TRα* and *TRβ* genes, is the thyroid hormone receptor gene (*TR*), which is crucial for both the maintenance and control of the body’s natural differentiation and development as well as the management of metabolic balance in vivo. The primary roles of *TRα* in the body are to control the body’s growth and development and preserve thyroid function. The primary roles of *TRβ* in the body are to maintain the negative feedback of thyroid hormone (*TH*) to thyrotropin (*TSH*) and to enhance the sensitivity of *T3*. This study examines the thyroid-interfering impact of fenpropathrin on *Gobiocypris rarus* by analyzing the expression of the *TRα* and *TRβ* genes following exposure. The findings are displayed in Figure 8.

Following 31 days of fenpropathrin exposure, there was a significant increase in the relative expression level of the *TRβ* gene in young *Gobiocypris rarus* in the 1.5 and 2.0 μg·L^−1^ concentration groups when compared to the control group. However, there was no significant difference in the relative expression level of the *TRα* gene between the concentration groups. The findings demonstrated that fenpropathrin disrupted the expression of the *TRβ* gene in bio-rare young fish, hence affecting the thyroid system of these fish. This work adds to the body of knowledge on the mechanism of fenpropathrin’s thyroid interference in *Gobiocypris rarus*; however, further research and supplementation are required to fully understand the regulatory mechanisms of pyrethroids on different components of the thyroid system.

### 3.6. Effects of Fenpropapathrin on Gene Expression of Aromatic Hydrocarbon Receptors in Gobiocypris rarus

Aromatic hydrocarbon receptor genes (*AHRS*) are ligand-induced transcription factors that can stimulate the expression of *CYP1A1* and *CYP1A2*, which are key immune system components. Additionally, they can participate in the response stress stimuli like radiation, infection, inflammation, and oxidative stress; they can also act in receptor-mediated toxic reactions to various environmental toxins (polycyclic aromatic hydrocarbon compounds); they can mediate a variety of cytotoxicity reactions; and they can play a role in important biological processes like signal transduction, cell differentiation, apoptosis, tumor evolution, growth and development, and regeneration.

**Figure 9:**
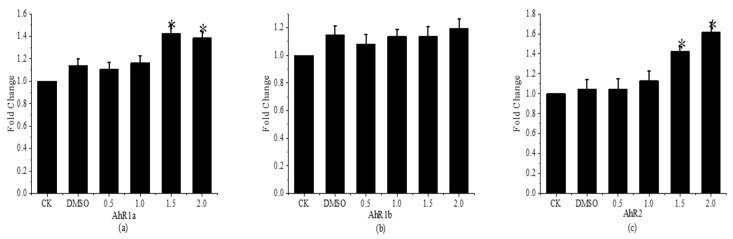
Relative expression of *AhRs* in young *Gobiocypris rarus* 31 days after fenpropathrin exposure. All data are shown as mean ± SE (n = 3), “*” indicate significant differences (*p* < 0.05). (**a**): Relative expression of *AhR1a*; (**b**): Relative expression of *AhR1b*; (**c**): Relative expression of *AhR2*.

Figure 9 presents the results of the expression of *AhRs* of fenpropathrin during development in juvenile fish. The findings demonstrated that, in comparison to the control group, the relative expressions of the *AhR1a* and *AhR2* genes in *Gobiocypris rarus* in the 1.5 and 2.0 μg·L^−1^ concentration groups were considerably higher, reaching 1.42 and 1.39, and 1.41 and 1.62 times, respectively. The control group’s relative expressions of the *AhR1a* and *AhR2* genes in *Gobiocypris rarus* were considerably lower in the remaining concentration groups. The two groups did not differ significantly from one another. The relative expression of the *AhR1b* gene in *Gobiocypris rarus* did not differ significantly between the concentration groups and the control group. In conclusion, juvenile *Gobiocypris rarus* exhibit altered *CYP* expression in vivo and altered metabolic levels due to a specific quantity of clathrin interfering with the expression of the *AHRS* gene.

## 4. Discussion

Pyrethroid pesticides are the third generation of pesticides following carbamate, organochlorine, and organophosphorus pesticides. They have been widely utilized in agriculture and their use is increasing every year due to their advantages of high efficiency, broad spectrum, minimal toxicity to mammals, and low residue. Fenpropathrin is one of the most commonly used pyrethroid pesticide types. Pyrethroid insecticides can infiltrate the aquatic environment in three different ways. The first is that pesticides used on agricultural land seep into the water table via surface runoff and sedimentation [23]. The second is that pesticides are used improperly, for example when pyrethroid pesticides are used in fish farming to clean ponds and end up directly in aquaculture water. The third is because human usage of mosquito repellent incense and mosquito repellent water residue from home sewage enters the water body and causes problems. Due to the widespread use of pyrethroid pesticides in recent years, they have been found in an increasing number of aquatic environments worldwide [4,5,6]. In addition, pyrethroid pesticides were detected in the Jiulong River Estuary [7], the Guanting Reservoir [8], the Liangtan River Basin [8], and the Zhujiang River network [9], among which the concentration range of fenpropathrin was ND~40.8 ng·L^−1^. The persistence of pesticides like fenpropathrin in the water environment can be harmful to fish because of their high toxicity to aquatic creatures. Research has demonstrated that fenpropathrin’s harmful effects on zebrafish at various developmental stages are caused by distinct mechanisms. Fenpropathrin primarily modulates the expression of genes involved in thyroid hormone production to alter the thyroid hormone balance in zebrafish during the development stage of their embryos and yolk sacs. Furthermore, fenpropathrin can induce a thyroid hormone imbalance in juvenile zebrafish by altering the expression of genes involved in thyroid metabolism and transformation during the growth stage of the fish [24]. Zebrafish embryo yolk sac development was negatively impacted by fenpropathrin exposure in a number of ways, including through teratogenic effects such as incubation inhibition, slowed heart rate, aberrant autonomic movement, and growth inhibition [25,26]. Fenpropathrin can also cause a considerable thyroid interference effect in zebrafish; by measuring the expression level of genes connected to the HPT axis, this impact was particularly noticeable in the juvenile fish’s growth stage. There was a considerable downregulation of *TRβ*, *Dio2*, *TG*, and *NIS* gene expression compared to in the other two embryonic stages. Fenpropathrin and other pyrethroid herbicides pose a serious risk to aquatic life. Fenpropathrin’s highly poisonous 96 h lethal concentration (LC_50_) for zebrafish is 3.375 × 10^−3^ mg·kg^−1^; a safe value is 0.3375 × 10^−3^ mg·kg^−1^. The induction effect of fenpropathrin at low concentrations for a brief period of time increased the activities of enzymes such as *GPT*, *GOT*, and *SOD*; however, at high concentrations and long-term exposure, the activities of these enzymes tended to decline. These effects were observed in the hepatopancreas of zebrafish exposed to the sublethal dose of fenpropathrin. Since *AChE* activity is highly sensitive to fenpropathrin, fenpropathrin poisoning results in a considerable reduction in enzyme activity, which is positively correlated with both the concentration of toxicants and the duration of exposure. *AChE* is a useful biological biomarker for tracking environmental contamination because of its sensitive response to environmental toxicants. Early in their development, zebrafish exhibited a robust resistance to fenpropathrin that was five times greater than that of adult fish. Indicators of the effects of environmental toxicants included death, sluggish heart rate, increased pericardial edema rate, reduced hatchability rate, and deformity created by spine curvature. Particular attention should be given to the amount of pesticide used, the application technique, and its impact on the aquatic ecology in agricultural production, particularly when applying pyrethroid pesticides like fenpropathrin in paddy fields. The use of pyrethroid insecticides in practical production is somewhat guided by the fact that, despite fenpropathrin’s extreme toxicity to fish in laboratory settings, its toxicity in natural water and soil can be greatly reduced due to enzymatic hydrolysis, adsorption, movement, and deposition [27].

The incubation of *Gobiocypris rarus* embryos was impacted by a minimum observable effect concentration (LOEC) of more than 2.5 μg·L^−1^. The unobserved concentrations (LOEC and NOEC) that had an impact on death and developmental deformity in *Gobiocypris rarus* larvae were 1.5 and 2.0 μg·L^−1^, respectively. After 31 days of fenpropathrin exposure, 1.5 μg·L^−1^ fenpropathrin exposure significantly increased the expressions of thyroid hormone receptor genes (*TRβ*) and aryl hydrocarbon receptor genes (*AhR1a* and *AhR2*) in juvenile *Gobiocypris rarus,* and significantly decreased the expressions of androgen receptor genes (*AR*) and sex hormone synthesis-related genes (*CYP17* and *CYP19a*). When exposed to 2.0 μg·L^−1^ of fenpropathrin, the expression levels of genes related to sex hormone synthesis (*HMGR*, *CYP17*, and *CYP19a*) as well as the androgen receptor gene (*AR*), estrogen receptor gene (*ER1*), and aromatic hydrocarbon receptor were downregulated, while the expression levels of genes related to *ER2a*, the thyroid hormone receptor gene (*TRβ*), and the estrogen receptor gene (*ER2*) were upregulated in juvenile *Gobiocypris rarus*. Reduced levels of fenpropathrin interfere with the reproductive system, thyroid system, and metabolic level in juvenile *Gobiocypris rarus* by altering the expression of genes linked to sex hormone synthesis, sex hormone receptor genes, and aromatic hydrocarbon receptor genes.

Androgen receptors (*AR*) and estrogen receptors (*ERs*) are examples of sex hormone receptors. These two receptor genes were chosen as study subjects in order to examine the impact of fenpropathrin’s reproductive endocrine disruption on the growth of *Gobiocypris rarus*. Fish gonads and brains are often where *AR* and *ERs*, which are members of the nuclear receptor superfamily, are expressed [28]. Studies that have previously been conducted have demonstrated that certain substances influence the production of nuclear receptors for the sex hormones, with effects on aquatic species similar to those of estrogen. For instance, endogenous estrogens are impacted by organochlorine pesticides, which bind to estrogens on target cells [28]. Pyrethroid pesticides can interfere with sex hormone synthesis and metabolism, and have an estrogen-like action, an antiandrogen effect, and other reproductive endocrine disruption mechanisms. Research on how pyrethroid pesticides interfere with estrogen-like and antiandrogenic effects has demonstrated that exposure to fenvalerate, permethrin, and beta-fenpropathrin suppressed the expression of *AR* in juvenile zebrafish, indicating a potential antiandrogenic effect. Regarding estrogen receptor genes, exposure to beta-fenpropathrin increased the expression of *ER1*, while *ER2a* was down-regulated in juvenile zebrafish; exposure to fenvalerate increased the expression of *ER1*, while exposure to permethrin increased the expressions of *ER2a* and *ER2b* [29]. Comparing fenpropathrin’s effects on *Gobiocypris rarus* in developing sex hormone receptor genes, this study also reveals that fenpropathrin inhibits the expression of both estrogen and androgen receptor genes at the genetic level.

Within the nuclear receptor superfamily, which is mostly composed of the *TRα* and *TRβ* genes, is the thyroid hormone receptor gene (*TR*), which is crucial for both the maintenance and control of the body’s natural differentiation and development and the management of metabolic balance in vivo. The primary roles of *TRα* in the body are to control the body’s growth and development and preserve thyroid function. The primary roles of *TRβ* in the body are to maintain the negative feedback of thyroid hormone (*TH*) to thyrotropin (*TSH*) and to enhance the sensitivity of *T3* [30,31,32]. Fish cannot grow or develop without the influence of the thyroid hormone since it can influence how fish embryos develop into young fish. Numerous investigations have demonstrated that endocrine disruptors can alter thyroid function in fish by interfering with the thyroid system’s ability to regulate blood flow. For instance, *T3* and *T4* in zebrafish are significantly inhibited by the flame retardant hexabromocyclododecane, and their content levels exhibit a declining trend as the exposure concentration increases [33]. Nano-copper oxide has the ability to alter the morphology of epithelial cells, harm thyroid follicular structure, raise thyroid hormones *T3* and *T4*, and suppress the expression of the gene *ugt1ab*, which codes for a metabolic enzyme [34]. Exposure to polychlorinated biphenyls (*PCBs*) can result in a marked drop in plasma thyroid hormone levels, as well as an evident expansion of thyroid follicular cells and glial abnormalities in juvenile flounder [34]. This study’s findings suggest that exposure to fenpropathrin at 1.5 μg·L^−1^ interferes with the expression of the *TRβ* gene in *Gobiocypris rarus*, which in turn interferes with the thyroid system of juvenile fish. This work adds to the body of knowledge regarding the genetic mechanisms underlying fenpropathrin’s interference with thyroid function in *Gobiocypris rarus*. Further research is still needed to determine the precise regulatory mechanism by which pyrethroids affect the thyroid system.

The immune system’s normal operation is supported by the ligand-induced transcription factors known as aromatic hydrocarbon receptor genes (*AHRS*). These can cause the expression of *CYP1A1* and *CYP1A2*; take part in inflammatory, oxidative, radiation, and other stress responses; and mediate a range of cytotoxic reactions and critical biological processes, including growth and development, signal transduction, cell differentiation, apoptosis, tumor evolution, and regeneration [33,34]. Exogenous pollutants can affect the expression of mammalian aromatic hydrocarbon receptor genes. According to existing studies on pyrethroid pesticides, exposure to fenpropathrin can affect the expression of rat *CYP1A*, thus affecting its oxidative metabolic function [35]. Research has indicated that certain external contaminants can also impact fishes’ expression of aromatic hydrocarbon receptors and obstruct their oxidative metabolism. For instance, ovarian aromatase gene expression can be inhibited by exposing female zebrafish to a low dose of terazosin, but aromatase gene expression can be enhanced by exposing female zebrafish to a high dose. Furthermore, testis aromatase in male zebrafish can be substantially inhibited by triazotin at all concentrations, according to the expression of the enzyme gene [36]. Dioxin-like polychlorinated biphenyls (*PCB126*) have a notable dose–effect relationship in zebrafish embryos, as they can considerably increase *EROD* activity and the relative expression level of *CYP1A* gene mRNA [37]. *CYP* has a protective effect on the metabolism of permethrin [38], and cypermethrin can mediate the transcription level and catalysis of the *CYP* enzyme in young and adult zebrafish [39]. According to this study’s findings, *Gobiocypris rarus*’s AHRS gene expression starts to be affected at 1.5 μg·L^−1^ clathrin exposure, which in turn affects *CYP* expression in vivo and the organism’s metabolic state.

## 5. Conclusions

Significant developmental defects, such as somatic curvature, body axis curvature, and yolk sac edema, may be induced by exposure to 1.5 and 2.0 µg·L^−1^ fenpropathrin for 7 to 9 days, although no significant influence was observed on embryo incubation (LOEC > 2.5 µg·L^−1^). The expression of genes related to sex hormone synthesis, thyroid hormone receptors, sex hormone receptors, and carbons in *Gobiocypris rarus* can be affected by 31 d of deltamethrin exposure to fenpropathrin with LOEC = 2.0 µg·L^−1^ and NOEC = 1.5 μg·L^−1^. This is based on the toxicity endpoints for the development and death of *Gobiocypris rarus* at early life stages. The thyroid and reproductive systems of fish are affected by the expression of receptor genes. There was an inhibitory effect of fenpropathrin on the androgen receptor gene. By interfering with the expression of the *TR* beta gene, fenpropathrin can interfere in the thyroid system in young *Gobiocypris rarus* fish. In *Gobiocypris rarus*, exposure to fenpropathrin at a concentration of 2.0 μg·L^−1^ can decrease the expression levels of genes related to sex hormone synthesis (*HMGR*, *CYP17*, and *CYP19a*), the androgen receptor gene (*AR*), the estrogen receptor gene (*ER1*), and the aromatic hydrocarbon receptor gene (*AhR1a* and *AhR2*). Conversely, the expression levels of genes related to thyroid hormone receptor (*TRβ*), the estrogen receptor gene (*ER2a*), and the thyroid hormone receptor gene (*TRβ*) were upregulated. Reduced levels of fenpropathrin interfere with the reproductive system, thyroid system, and metabolic level of *Gobiocypris rarus* by altering the expression of genes linked to sex hormone synthesis, sex hormone receptor genes, and aromatic hydrocarbon receptor genes.

## Figures and Tables

**Table 1:** Primer sequences of real-time quantitative PCR.

Gene Name	Primer Sequences	
	Positive	Reverse
*β-actin*	TGCTGTTTTCCCCTCCATTG	TCCCATGCCAACCATCACT
*AR*	TCTGGGTTGGAGGTCCTACAA	GGTCTGGAGCGAAGTACAGCAT
*ER1*	GGTCCAGTGTGGTGTCCTCT	CACACGACCAGACTCCGTAA
*ER2a*	AGCTTGTGCACATGATCAGC	GCTTTCATCCCTGCTGAGAC
*ER2b*	TTGTGTTCTCCAGCATGAGC	CCACATATGGGGAAGGAATG
*TRα*	CAATGTACCATTTCGCGTTG	GCTCCTGCTCTGTGTTTTCC
*TRβ*	TGGGAGATGATACGGGTTGT	ATAGGTGCCGATCCAATGTC
*AhR1a*	CGCAAAAGGAGGAAACCTGTC	CCTGTAGCAAAAATTCCCCCT
*AhR1b*	GGAGAGCACTTGAGGAAACG	GGATCCAGATCGTCCTTTGA
*AhR2*	ATCTCCATGGGCAAAACAAG	TCCCTCTTGTGTCGATACCC
*HMGR*	CAAAGCACATCCCATCTTACAAAC	TTCCCATCACCATAGAGTAGTCGTA
*StAR*	TTGTAAGTGTCCGCTGTGCCA	GCATCACAATACAGGTGGGTCC
*3β-HSD*	TCCACACAGCGTCTCTCATCG	TGGGACCAGCCACCTCAATG
*CYP17*	TCTCCGCTCCTCATCCCTCA	CACAAACCATCACCCTCCTCATT
*CYP19a*	TCGTTTCTTTCAGCCGTTCG	TGCTGCGACAGGTTGTTGGT

## Data Availability

The data presented in this study are available upon request from the corresponding author.
